# Mechanical Properties and Mechanism Analysis of Graphite Tailings Environment-Friendly Concrete

**DOI:** 10.3390/ma15248870

**Published:** 2022-12-12

**Authors:** Hourui Duan, Hongbo Liu, Bochen Li, Zhongrui Wang, Hongshuai Gao

**Affiliations:** 1 School of Civil Engineering, Heilongjiang University, Harbin 150080, China; 2 Key Laboratory of Functional Inorganic Material Chemistry, Heilongjiang University, Ministry of Education, Harbin 150080, China

**Keywords:** concrete, environment-friendly, graphite tailings, mechanism analysis, mechanical properties

## Abstract

The development of tailings in concrete technology is not only conducive to the realization of the goal of reducing carbon emissions, but also conducive to the inhibition the occurrence of shortages of sand and gravel supplies. In this study, graphite tailings were used to replace sand in the range of 0~100%, and the mechanical mechanism of graphite tailings concrete was examined through compressive and flexural tests. The mechanical experimental results were evaluated and verified based on concrete macroscopic failure appearance, mesoscopic failure appearance, and physical characteristics of graphite tailings. The results revealed that the concrete strength increases first and then decreases with the increase of the graphite tailings content. Compared to GT00 (GT00 is a specimen with a graphite tailings content of 0%, and so on), GT10~GT60 exhibited better mechanical properties, of which 30% was recommended as the optimal replacement rate. The mechanical properties of GT10 and GT20 had an upward trend, and GT30 had low spalling, with aggregate fragmentation found on the fracture surface. GT30 showed the best resistance to bending and deformation. The mechanical properties of GT40~GT60 had a downward trend. When the graphite tailings content was higher than 70%, the interface defects of the aggregate matrix increased, thus making it easier for cracks to propagate along the interface. Furthermore, the mechanism of graphite tailings replacing sand verified the test results from different perspectives, which provides new analysis ideas for other tailings in environment-friendly concrete.

## 1. Introduction

Concrete is an essential material for the construction of modern industrialized society and has been extensively used in all walks of life [1,2,3]. It takes on a critical significance in energy systems, water and sewage systems, high-rise buildings, as well as transportation networks. Countries worldwide are committed to modifying laws and policies about carbon reduction and low carbon, simplifying the certification and promoting the application of green building materials, and deepening the research of sustainable building materials (e.g., new cementitious materials, environment-friendly concrete, and wood and bamboo building materials). The above concepts have gradually become the focus of the construction industry [4,5,6,7,8,9]. The concrete industry, one of the largest CO_2_ emission industries, can boost the sustainable development of concrete by reducing carbon emissions in each link in its entire life cycle [10,11]. Moreover, the international community is advocating a low-carbon economy, so the environment-friendly development of sustainable concrete is a vital direction for the concrete industry. Scholars worldwide have extensively studied low-carbon concrete [5,12,13,14], whereas its practical application is still limited. Combing the carbon reduction methods of the concrete industry and preparing environment-friendly concrete takes on a great significance in the world’s goal of reducing CO_2_ emissions.

Tailings are the general term for the remaining waste after the ore is crushed and sorted, and they are one of the essential types of industrial solid waste. On one hand, the accumulation of tailings will cause environmental pollution through sand blowing or seepage [15]. On the other hand, the tailings will produce CO_2_ during the treatment process [16], so the treatment of tailings is very important to the global ecological process. Countries with mineral resources and mineral processing produce considerable tailings, thus making the discharge of tailings inevitable. The problems caused by tailings have aroused a growing attention from the public towards the continuous increase of the scale of tailings [17,18]. Since the 20th century, some mining enterprises have committed to the development and utilization of tailing resources for resource depletion, environmental protection, and employment solutions. Some applications have been achieved, including the recovery of valuable metals and non-metal elements in tailings [19], the production of building materials from tailings [20], as well as the use of magnetized tailings as soil conditioners [21]. Since the main components of tailings (e.g., copper, iron, antimony, and molybdenum) are sand and gravel, the use of such tailings as aggregates for concrete has been considered a vital development direction of tailings utilization. Molybdenum tailings are similar to sand in density, microstructure, and chemical composition [22], and molybdenum tailings can achieve a higher replacement rate (100% replacement rate) when molybdenum tailings serve as fine aggregates to prepare environment-friendly concrete components [23]. The compactness and waterproofing properties of concrete are improved when iron tailings replace sand [24], and when preparing environment-friendly concrete from iron tailings, the autogenous shrinkage rate of iron tailings concrete was 9.8% higher than that of sand concrete at the age of 90 days [25]. Copper tailings can refine the pore size and promote long-term strength development of concrete [26], and replacing sand with copper tailings is conducive to the utilization of copper tailings and the improvement of concrete mechanical strength on the premise that the mechanical strength of concrete meets requirements. Li et al. [27] prepared ecological concrete using antimony tailings as aggregates, and the results revealed that high-strength concrete at different substitution rates meets the requirements of industry standards. Otieno et al. [28] used kimberlite tailings as aggregates in concrete and suggested that the addition of kimberlite tailings reduced the compressive strength and durability of concrete, whereas appropriate mix ratio and admixture could make up for the negative effects of kimberlite tailings.

The application of tailings as fine aggregates in concrete has been proven. As a kind of tailing, graphite tailings (GT) have irreversible effects on the environment, and the research on GT is in the initial stage. Currently, there are many gaps in the research on GT, and some issues are worth noting, including not knowing the optimal replacement rate of GT, whether they can completely replace sand as fine aggregates, and the application mechanism of GT in concrete. In this paper, the influence mechanism of GT in concrete is analyzed based on the basic physical properties of GT. First, GT and sand were compared to analyze the feasibility of replacing sand with GT. Subsequently, the compressive and flexural tests were performed, and the damage characteristics of GT environment-friendly concrete were analyzed in accordance with the experimental result. Lastly, GT ecological concrete was studied from four aspects, including mechanical properties, macroscopic cracks, mesoscopic appearance, and the study on the mechanism of GT substituting sand (the cracks and spalling of concrete were taken as the macroscopic analysis object, the mesoscopic appearance was the mesoscopic analysis object, and the mechanism analysis of the results of GT replacing sand was conducted from different perspectives).

## 2. Experimental Programs

### 2.1. Materials

#### 2.1.1. Cement

**Figure 1:**
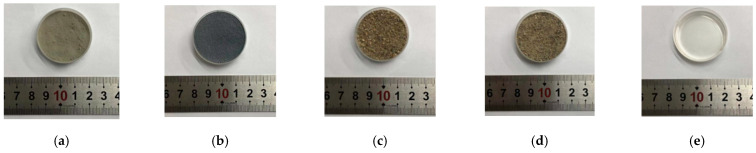
Materials used in the production of concrete. (**a**) Cement; (**b**) silica fume; (**c**) sand; (**d**) graphite tailings; (**e**) superplasticizer.

Portland cement (P.O. 42.5 N) was the cement used in this study, with a density of 3.0 g/cm^3^ and a specific surface area of 380 cm^2^/g. The chemical composition of the cementitious material is determined according to the Chinese standard (GB/T 176-2017) [29], the chemical composition is listed in Table 1, and the picture is presented in Figure 1a.

#### 2.1.2. Silica Fume

The silica fume was provided by Xinming New Building Materials Co., Ltd., Heilongjiang, China. Table 1 lists the chemical composition of the silica fume. As depicted in the table, the silica fume was primarily made up of SiO_2_ (92.8%). Figure 1b presents the picture of silica fume.

#### 2.1.3. Aggregate

Sand was used as fine aggregates, and the maximum particle size of the sand applied was 2.5 mm. The GT sample was obtained from a graphite mine in Jixi City, China, and the maximum particle size of GT was 1.25 mm. Figure 1c,d illustrate the pictures of sand and GT, respectively.

#### 2.1.4. Admixture

A polycarboxylate-based superplasticizer was provided by Tianjin Weihe Technology Development Co., Ltd., and the water reducing rate of the admixture was 30%. Figure 1e depicts the picture of the admixture.

### 2.2. Feasibility Analysis of Replacing Sand with GT

#### 2.2.1. Physical Property Analysis

The basic physical properties of tailings are the prerequisites for the mechanical properties of environment-friendly concrete to meet requirements [30]. The physical properties of aggregate were examined in accordance with Chinese standards for technical requirements and test methods of sand and crushed stone (or gravel) for ordinary concrete (JGJ 52-2006) [31]. Table 2 lists the basic physical parameters of GT and sand. As depicted in this table, the bulk density of sand was slightly larger than that of GT, the apparent density of sand was slightly smaller than that of GT, and there was a slight difference between the bulk density and apparent density of the two. The fineness modulus of sand was larger than that of GT, thus suggesting that the average particle size of sand was larger than that of GT, the skeleton of sand in concrete was better than that of GT, and GT with a small particle size could fill the sand. The crush rate of sand was less than that of GT, thus suggesting that GT are more easily crushed than sand, which leads to the decrease of the concrete strength. The porosity of GT was larger than that of sand, and concrete compaction may be affected in concrete when the GT dosage is high. The water absorption rate of GT was higher than that of sand. The greater the water absorption of the aggregate, the less the effective the water–cement ratio participating in the hydration reaction will be, thus hindering the hydration reaction of the cementitious material.

#### 2.2.2. Chemical Composition Analysis

The chemical composition of the aggregate is determined according to the Chinese standard (JC/T 1084-2019) [32]. The main components of sand were SiO_2_, CaO, and Al_2_O_3_, and sand also contained a small amount of Fe_2_O_3_, MgO, K_2_O, and other substances (Table 3). The content of SiO_2_ was the largest, accounting for nearly 70% of the total material content, and CaO and Al_2_O_3_ accounted for 8.27% and 7.39%, respectively. The main components of GT were composed of SiO_2_, CaO, Al_2_O_3_, and Fe_2_O_3_. In addition, GT contained a small amount of MgO, K_2_O, and other substances (Table 3), of which SiO_2_ had the largest content (56.64%). The contents of CaO, Fe_2_O_3_, and Al_2_O_3_ were 13.34%, 7.39%, and 11.52%, respectively. The material contents of sand and GT were similar, and the main chemical component was SiO_2_, thus laying a basis for GT to replace sand in terms of material composition.

#### 2.2.3. Particle Size Analysis

**Figure 2:**
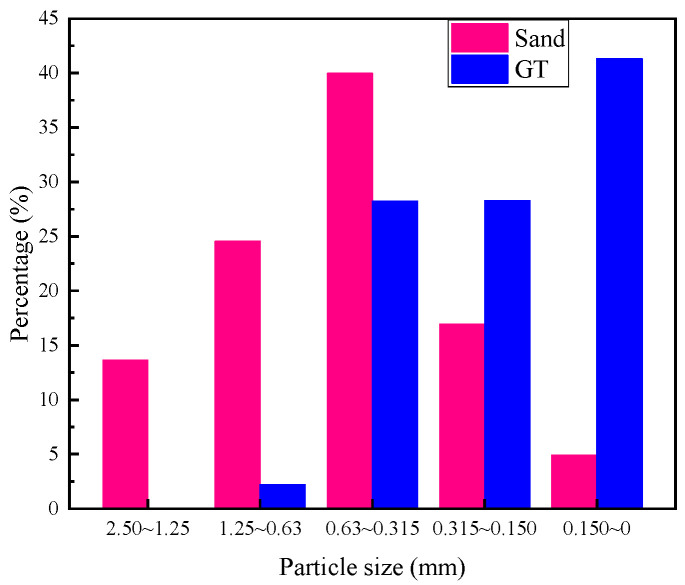
Particle size distribution of sand and GT.

The particle size distribution is determined according to the Chinese standard (JGJ 52-2006) [31]. As shown in Table 4 and Figure 2, the particle size distribution of GT was similar to that of sand, and both were between 2.5 and 0 mm. The main particle size distribution range of sand was 1.25~0.315 mm, accounting for over 60% of the total particle size. The particle size distribution range of GT was primarily 0.63~0 mm, accounting for more than 90% of the total particle size. The common particle size distribution range of GT and sand was 0.63~0.315 mm, and there was a solid basis for the particle size range of GT substituting sand.

#### 2.2.4. Comparison of GT and Sand Mesoscopic Pictures

Aggregates of different roughness will have different effects on the microstructure of concrete [33,34]. Table 5 presents the mesoscopic pictures of GT and sand with different particle sizes. As depicted in Table 5, sand particles were relatively smooth, and the surfaces of GT particles were rough.

Some researchers have investigated GT to improve rubber strength, prepare ceramic tiles, and use as a cementitious material [18,35,36]. There has been rare research on GT environment-friendly concrete. Liu et al. [37,38,39,40,41,42] prepared GT concrete with high electrical conductivity, frost resistance, and mechanical properties. Kathirvel, P. et al. [43] suggested that the optimal mechanical properties and durability can be obtained when GT are replaced by 40% volume content of sand. As revealed by the above discussion, it is feasible to replace sand with GT. In this study, the replacement of sand by GT to prepare ecological concrete was investigated, and the test scheme and results are presented as follows.

### 2.3. Test Mix Ratio

Volume replacement was used as the replacement method for GT replacing sand, and there were 11 groups from 0% to 100%. Table 6 lists the final proposed mix ratio of the test. GT are the only variable and the contents of water, cement, silica fume, and superplasticizer are kept constant for all samples.

### 2.4. Concrete Preparation Process

**Figure 3:**
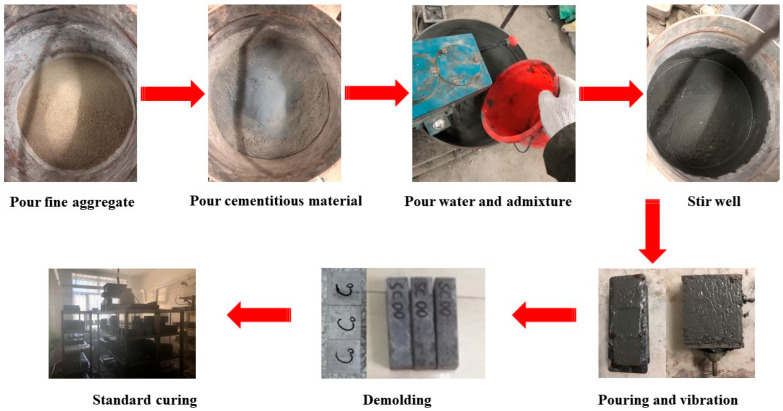
Concrete preparation process.

The preparation process of GT environment-friendly concrete is as follows, and Figure 3 illustrates the preparation steps.

(a) Pour GT and sand into the mixing pot and stir at a low speed for 3 min (the aggregates of different particle sizes were filled with each other, and finally mixed evenly).
(b) Pour the cement and silica fume into the mixing pot and stir at a low speed for 3 min (the aggregate was further filled with cementitious material, and the fully mixed raw materials contributed to the hydration reaction).
(c) Pour the pre-mixed water–admixture mixture into the stirring pot and stir at a low speed for 2 min, then stir at a high speed for 3 min (hydration and pozzolanic reactions are carried out in the stirring pot).
(d) Pour the concrete slurry into molds of 70.7 mm × 70.7 mm × 70.7 mm and 40 mm × 40 mm × 160 mm, and then put the molds on the vibrating table to vibrate for 7 min (the escape of air bubbles from the concrete increases the density of the concrete).
(e) Place the vibrated concrete slurry in the environment of ambient temperature 20 ± 1 °C and relative humidity of 60% for 24 h (create a good curing environment for the concrete).
(f) Demold the formed concrete and perform standard curing in a curing room at 20 ± 2 °C and a relative humidity of ≥95%, the specimens are cured for 3, 7, and 28 days (concrete hardening molding).

### 2.5. Test Methods

#### 2.5.1. Compressive Strength Test

**Figure 4:**
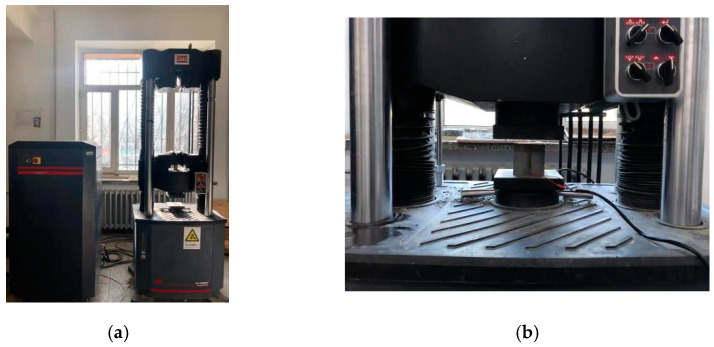
Compression test. (**a**) Compressive test machine; (**b**) compressive strength test.

A compressive strength test was performed in accordance with the Chinese standard [44]. The test was carried out using an electronic universal testing machine with a capacity of 600 kN. The machine had a loading rate of 2400 N/s. Figure 4 illustrates the setup of the compressive test, and compressive strength can be calculated by Equation (1).

(1) $$f_{cc}=\frac{F}{A}$$

where $f_{cc}$ denotes the compressive strength of concrete (MPa); *F* represents the load when the specimen is broken (N); *A* expresses the area of the specimen under pressure (mm^2^).

#### 2.5.2. Flexural Test

**Figure 5:**
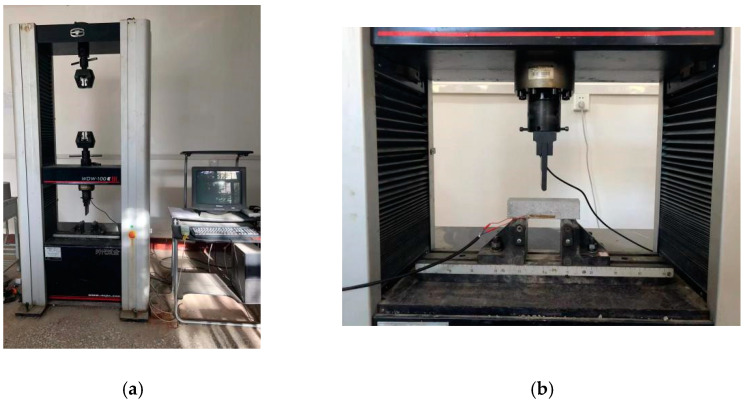
Flexural test. (**a**) Flexural test machine; (**b**) flexural test.

A flexural test was performed in accordance with the Chinese standard [45]. The test was conducted using a microelectronic control electronic universal testing machine with a capacity of 100 kN. The loading rate of the machine was 50 N/s. Figure 5 illustrates the setup of the flexural test, and flexural strength can be calculated by Equation (2).

(2) $$f_{f}=\frac{3Fl}{2b^{3}}$$

where $f_{f}$ denotes the concrete flexural strength (MPa); *F* expresses the load when the specimen is broken (N); *l* represents the span between supports (mm); *b* is the side length of the square section (mm).

## 3. Results and Discussion

### 3.1. Test Phenomenon

#### 3.1.1. Compression Test Phenomenon

**Figure 6:**
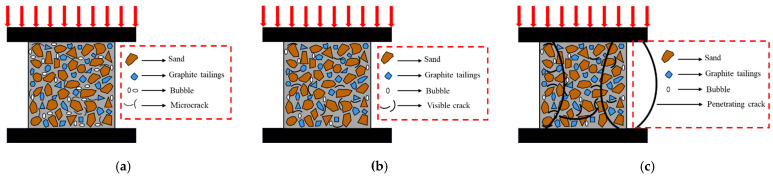
Schematic diagram of the compression stage of the specimen. (**a**) Compression stage of cracks and pores; (**b**) crack initiation stage; (**c**) crack propagation stage.

No obvious phenomenon was found when specimens were compressed at the beginning, whereas GT00~GT100 showed different degrees of peeling, which is to be analyzed in depth in Section 3.3.1. The concrete on the surface of the specimen gradually fell off with continuous application of load, and finally, accompanied by a “bang” sound, the specimen was crushed, and the test ended. The compression test piece had three stages.

(I) Compression stage of cracks and pores. The concrete was strengthened, and its corresponding microstructure changed. Some pores and cracks perpendicular to the force direction were compacted, and the concrete matrix was elastically compressed and more compact (Figure 6a).

(II) Crack initiation stage. The tip of the contact between the aggregate and cement stone, the micro-cracks within the concrete, and the places with pores within the concrete were prone to stress concentration under the application of load. Moreover, a part of the original cracks within the concrete began to extend or propagate, although they were all short and small in width. As the above microscopic cracks were extended or propagated, the stress concentration was relieved, and the equilibrium was restored immediately. The stress–strain was basically close to the linear elastic relationship at this stage (Figure 6b).
(III) Crack propagation stage. First, the existing cracks were further extended or propagated. Some cracks penetrated the mortar, some short cracks were connected to each other to form long cracks, and new cracks were generated at the same time. Subsequently, the number of continuous penetrations of the cracks increased sharply, and the crack development was accelerated. Lastly, the concrete was penetrated by cracks, and damage occurred (Figure 6c).

#### 3.1.2. Flexural Test Phenomenon

**Figure 7:**
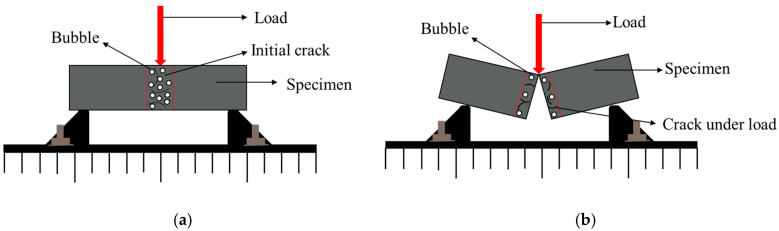
Schematic diagram of the bending resistance stage of the specimen. (**a**) Reinforcement stage; (**b**) destruction stage.

The flexural phenomena of the specimens with different GT contents were nearly the same, whereas the fracture surface after concrete failure exhibited a different meso-structure, which is to be analyzed in depth in Section 3.3.2. No obvious phenomenon was found in the process of the test piece being stressed, and the test was stopped when the specimen reached brittle fracture. The flexural resistance of the specimen was divided into two stages.

(I) Reinforcement stage. The load–displacement curve was concave at this stage, the pores and some micro-cracks within the concrete were compacted, and the stiffness of the specimen in the flexural section increased (Figure 7a).

(II) Destruction stage. The load–displacement curve of the specimen showed linear elastic development at this stage, the force applied to the concrete led to new cracks, and the rapid propagation of the cracks caused brittle failure of the specimen (Figure 7b).

### 3.2. Mechanical Property Analysis

#### 3.2.1. Analysis of Compression Test Results

I. Strength analysis

**Figure 8:**
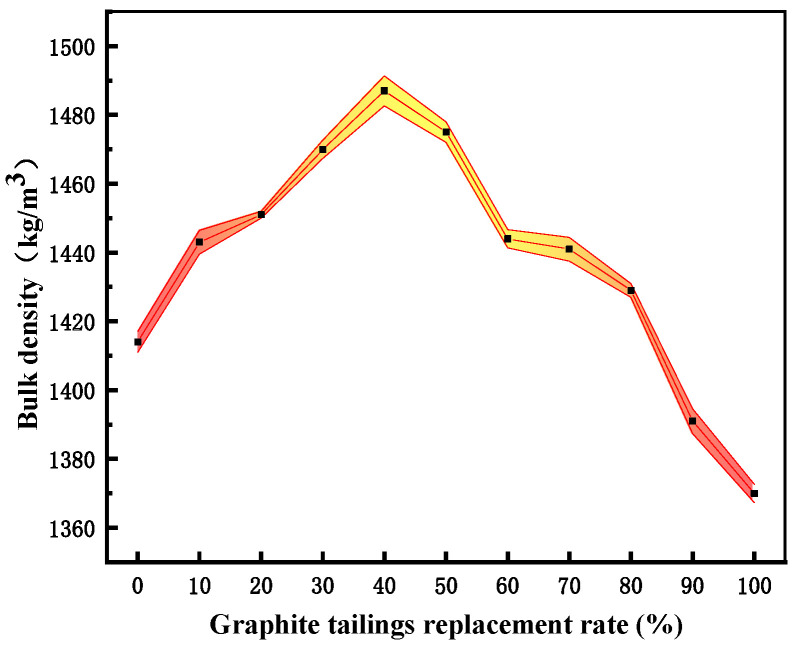
Bulk density of GT substitute sand.

**Figure 9:**
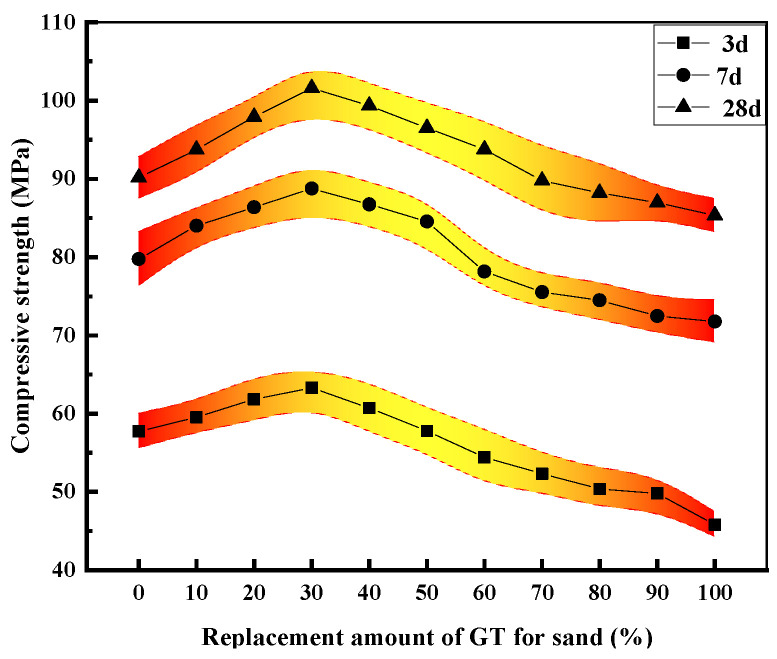
Compressive strength of specimens with different substitution rates.

As depicted in Figure 8 and Figure 9, the effect of GT on the aggregate bulk density was similar to that on the compressive strength, and the bulk density (compressive strength) increased with the increase of the GT dosage up to a limiting value of 40% (30%). The GT dosage increasing beyond the limiting value would lead to the reduction of the bulk density (compressive strength). However, the GT substitution rates corresponding to the maximum bulk density and the maximum compressive strength were different. GT has a small particle size and has a filling effect on sand, so a small amount of GT can replace sand to increase the bulk density of a GT–sand system. However, excessive GT will make the gradation of the GT–sand system uneven, and the filling effect will gradually be lost. The differences between GT and sand crushing values, water absorptions, and other properties makes the optimal replacement rate of GT change in concrete.

The compressive strength of GT30 was 12.68% higher than that of GT00. The strength of the specimen had the minimum value when GT replacement rate was 100% (the strength was reduced by 5.37%). The extreme values of the intensity of the 3-day, 7-day, and 28-day specimens were 17.5, 17.0, and 16.3, respectively, thus suggesting that the range of the specimen decreases with the curing age. As depicted in Figure 9, the compressive strength of the GT00 specimen was similar to that of GT50 when the curing time was 3 days. As the curing time reached 7 days, the compressive strength of GT00 was between GT50 and GT60, and the strength of GT00 was similar to GT70 at 28 days. Cement hydration and pozzolanic reaction of silica fume have been found as important factors for the mechanical properties and durability of concrete [46]. In the case of a low water-to-binder ratio, the packing effect of tailing particles cannot make up for the decrease in the effective amount of hydration of the cementitious material [47]. The water molecules in the curing room continue to enter concrete when concrete is cured, thus making the hydration reaction of the cementitious material proceed slowly with curing time. In general, the filling effect between aggregates, curing age, and curing conditions significantly affects concrete strength.

II. Stress strain analysis

**Figure 10:**
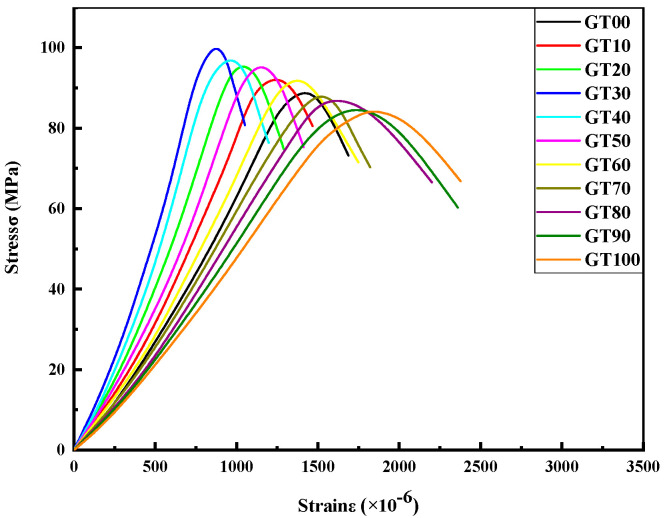
Compressive stress–strain curve.

Stress and strain indicate the internal stress process of concrete, and the mutual coordination relationship of each component under load is also reflected [48,49]. As depicted in Figure 10, the stress–strain trends of GT00~GT10 were the same, which were divided into two parts, including the ascending section and the descending section. Concrete stress increased slowly, and the strain increased rapidly at the initial stage of loading. The stress–strain curve was close to a straight line as the load continued to be applied, and the slopes of the GT00~GT100 curves were different since different GT contents made the concrete densities different. When approaching the peak stress, the slope of the curve to the left of the peak stress decreased, and the strain growth rate tended to be greater than the stress growth rate. Furthermore, the slope became 0 when the curve reached the peak stress. In the second half of the peak stress, the stress–strain curve began to decrease, the slope of the descending segment increased rapidly, and the specimen exhibited obvious brittle failure characteristics.

**Figure 11:**
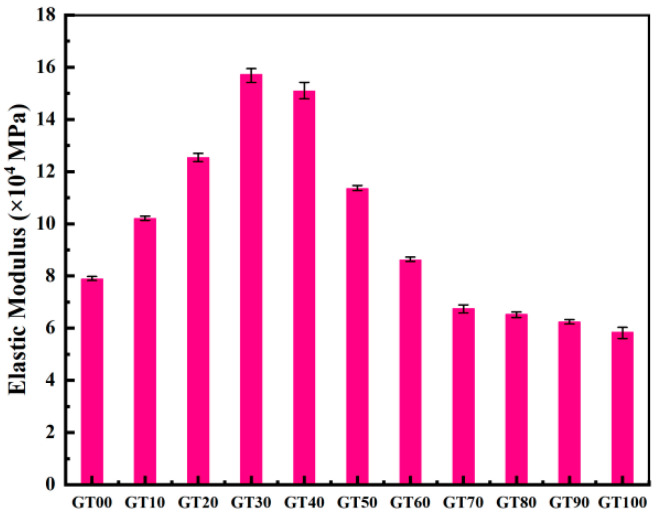
Elastic modulus.

The elastic modulus of GT00~GT100 was obtained in accordance with the stress–strain curve (Figure 11). There were two aspects to the effect of GT content on the elastic modulus of concrete. When the amount of GT added was small, the failure mechanism of concrete was that the internal micro-cracks deteriorated with the increase of load. The GT–sand system was relatively dense, and the rough surface of GT could have made the aggregate system have greater friction, so the increase of the elastic modulus of concrete was large and obvious. The elastic modulus of GT30 showed the most obvious increase (96.42% higher than that of GT00). When considerable GT were added, the compactness of the concrete structure decreased, and the high water absorption rate of GT made the effective water–cement ratio in the concrete hardening process smaller. To be specific, the elastic modulus of GT100 showed the most obvious decrease (25.96% lower than that of GT00). Notably, the increase of the elastic modulus was more pronounced than the decrease. The influence of tailings on the elastic modulus of concrete is multi-faceted [50]. The amount of GT added in concrete affects the compressive strength, porosity, and bond strength between the aggregate and matrix, thus changing the elastic modulus of concrete [51]. The surface roughness, particle size distribution, and crushing value of GT may affect the elastic modulus of concrete, which should be studied in depth.

#### 3.2.2. Analysis of Flexural Test Results

I. Flexural strength and load–displacement curve analysis

**Figure 12:**
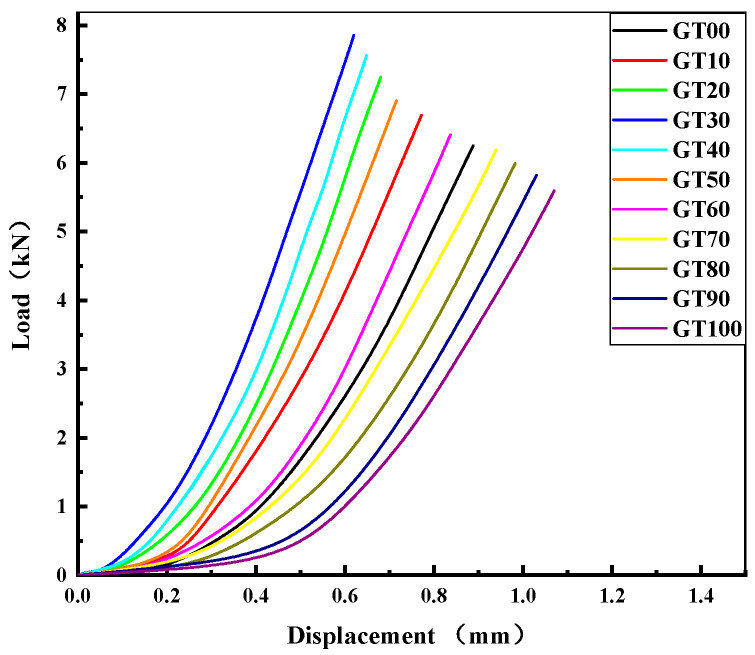
Load–displacement curve.

**Figure 13:**
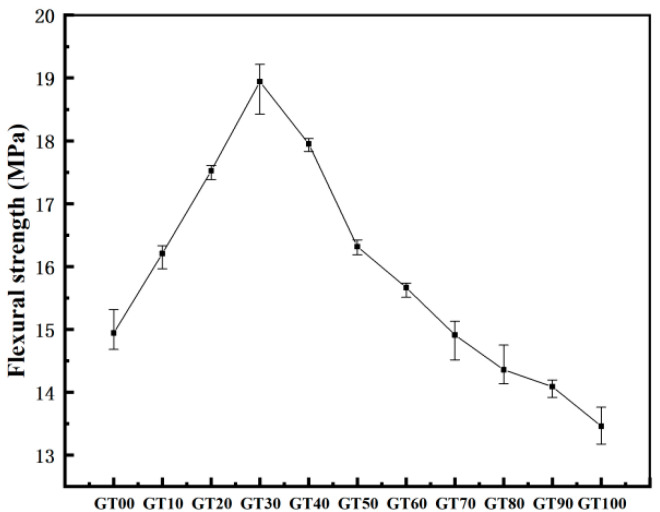
Flexural strength.

Figure 12 illustrates the load–displacement curve of the specimens with different GT contents. As depicted in Figure 12, the load–displacement curve was divided into two stages, including a strengthening stage and a linear elastic failure stage. At the strengthening stage, the slope of the curve tended to increase, and the flexural section stiffness increased continuously. At the linear elastic failure stage, the load–deflection curve developed in a linear elastic way until the specimen failed. Compared to GT00, when the GT content ranged from 10% to 60%, the strengthening time of the specimen was shorter and the deflection was smaller, thus suggesting that a small amount of GT can strengthen the concrete and increase the stiffness and flexural strength of the specimen (Figure 13), which is consistent with the existing analysis results. With the increase of the GT content, the duration of the strengthening stage of the specimen increased, the duration of the linear elastic failure stage decreased, and the peak load at the failure of the specimen also decreased (Figure 13). The maximum deflection values of GT90 and GT100 were higher than 1 mm and increased by 15.73% and 20.22%, respectively, compared to the deflection of GT00.

(3) $$R_{B}=\frac{3LP_{B}}{2bh^{2}}$$

(4) $$\varepsilon_{B}=\frac{6hd}{L^{2}}$$

(5) $$S_{B}=\frac{R_{B}}{\varepsilon_{B}}$$

where *b* denotes the width of the cross-interrupt interview piece (mm); *h* represents the height of the cross-interrupt interview piece (mm); *L* expresses the span of test piece (mm); *P_B_* is the maximal load when the specimen fails; *d* represents the mid-span deflection when the specimen fails; *R_B_* is the bending and tensile strength when the specimen is broken (MPa); *ɛ_B_* denotes the maximal bending and tensile strain when the specimen fails (με); *S_B_* expresses the flexural modulus when the specimen is broken (MPa).

**Figure 14:**
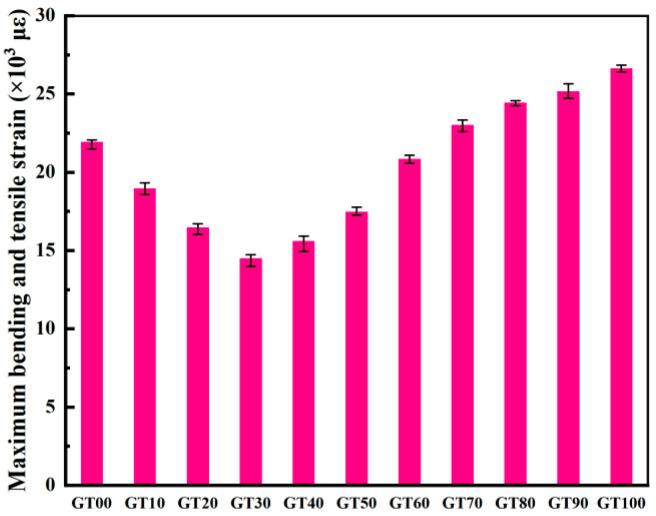
Maximum bending and tensile strain.

**Figure 15:**
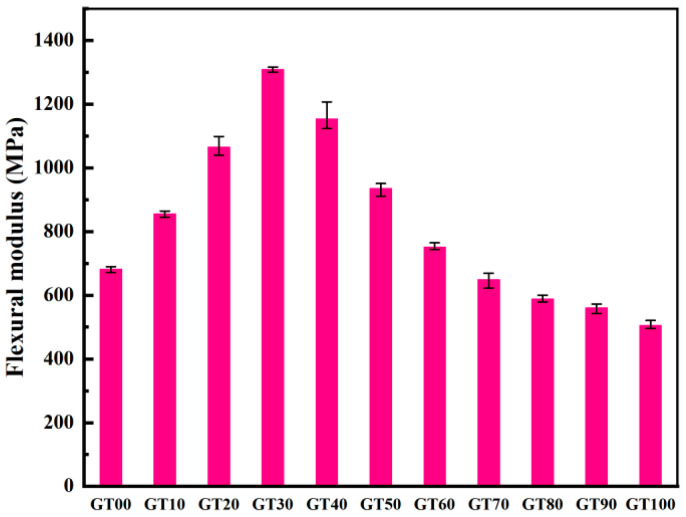
Flexural modulus.

The flexural modulus is capable of evaluating the ability of concrete to resist flexural deformation. According to Equations (3)–(5), the maximum flexural tensile strain (Figure 14) and flexural modulus (Figure 15) of concrete can be calculated. The GT–sand aggregate system exhibits certain elastic characteristics, and the incorporation of GT within a certain range can absorb the energy generated in the process of crack propagation, resulting in an increase of the flexural modulus of concrete. As depicted in Figure 15, 10~60% GT content had different degrees of contribution to the ability of concrete to resist deformation. The GT contents of 30% and 60% had the largest and smallest contributions to the flexural modulus of concrete (83.38% and 8.68%), respectively. There were more pores in the concrete when the GT content exceeded 70%, and the stress concentration generally occurred at the interface between the pores, the aggregate, and the matrix. As a result, the flexural modulus of the concrete was lower than at GT00. Although concrete has good ecological properties, its mechanical strength cannot be ensured at this time.

II. Stress strain and energy analysis

**Figure 16:**
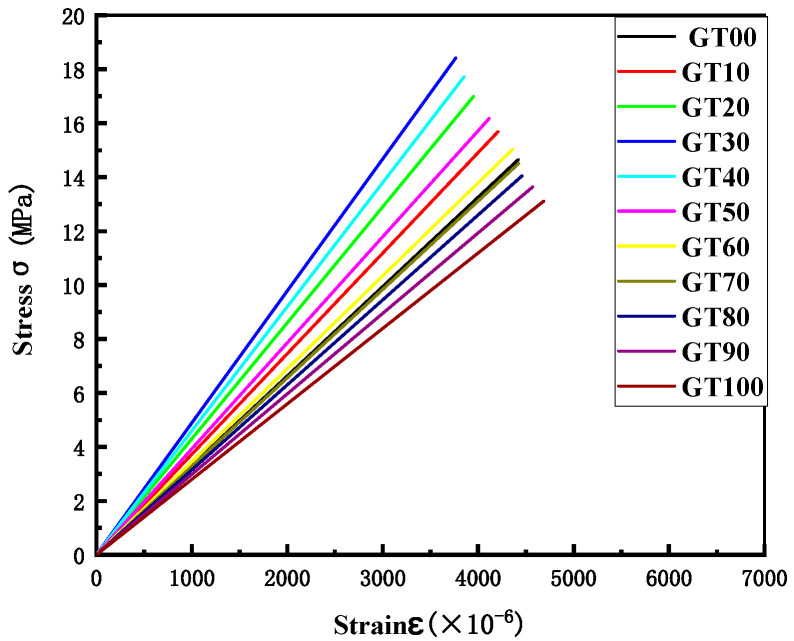
Stress–strain curve.

**Figure 17:**
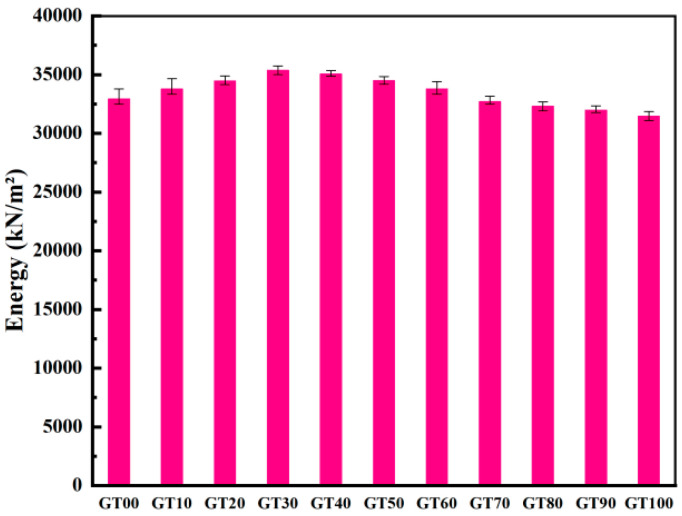
Energy required for specimen failure.

The crack propagation direction is perpendicular to the stress direction during the period of concrete stress, and the effective bearing area of the section tends to decrease. To consume less energy, the cracks will bypass the aggregate and propagate in the interfacial transition zone and the initial micro-cracks in the matrix. As depicted in Figure 16, the stress–strain curves under different GT contents were all linear elastic. The area enclosed by the stress and strain was the energy required for the concrete to resist fracture (Figure 17). The difference in the path makes the difficulty of the crack propagation different [52].

#### 3.2.3. Environment-Friendly Evaluation of GT Concrete from a Mechanical Point of View

Compared to GT00, GT30 exhibits the best mechanical properties, and 60% GT can ensure that the mechanical strength of concrete meets the requirements and the ecological properties reach the maximum. However, in practical engineering applications, the ecology of concrete may be changed by dimensional effects.

### 3.3. Macroscopic and Mesoscopic Analysis of GT Concrete

The propagation of concrete cracks was limited at the initial stress, and the concrete material absorbed a lot of external energy. Compared to GT00, the change range of fracture energy of GT10~GT100 was −5.09% (GT100)~7.13% (GT30). An appropriate amount of GT can inhibit the formation of initial micro-cracks in concrete, reduce the path of crack propagation in concrete, and prevent the dissipation of concrete energy. There were more defects in the matrix when GT replaced most of the sand, which was confirmed by mesoscopic analysis (Section 3.3.2). The deformation caused by the load should be released along with the increase of concrete strain, and brittle fracture of concrete occurs due to the absence of additional tensile stress.

#### 3.3.1. Analysis of Macroscopic Cracks and Spalling Degree

Concrete refers to a multi-phase composite material composed of aggregate and hardened cement paste, and as such, defects such as pores, capillary pores, and material cracks inevitably exist in the process of mixing and vibration molding. Loading causes concrete stress to develop, limiting its volume change, and the concrete specimen will crack once the tensile stress exceeds the tensile strength of the concrete [53]. Concrete eventually breaks down completely as the cracks converge and propagate repeatedly [54]. The appearance of concrete after failure can directly reflect the advantages and disadvantages of concrete mechanical properties.

**Figure 18:**
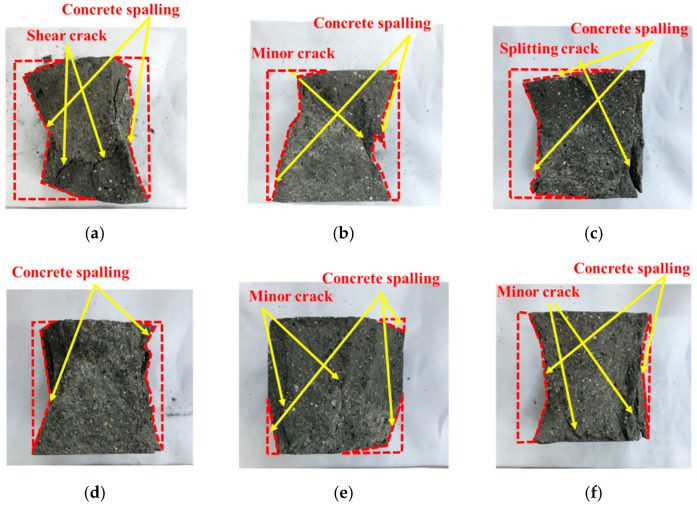

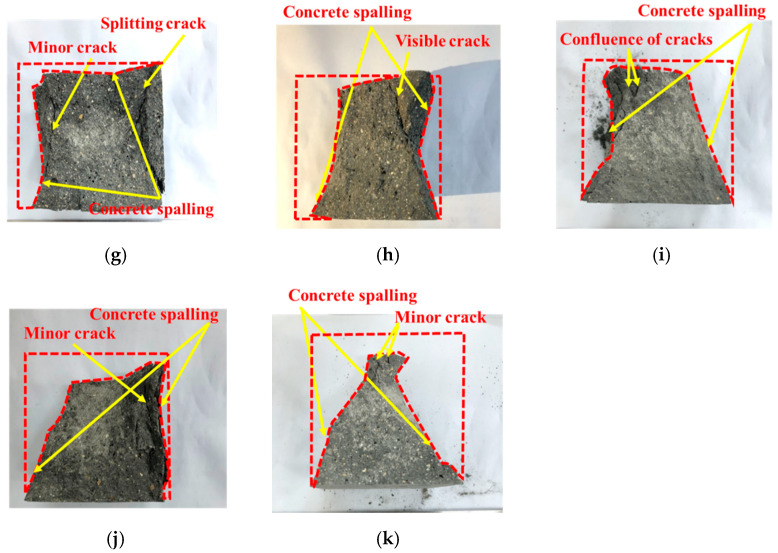
Failure patterns of different specimens. (**a**) GT00; (**b**) GT10; (**c**) GT20; (**d**) GT30; (**e**) GT40; (**f**) GT50; (**g**) GT60; (**h**) GT70; (**i**) GT80; (**j**) GT90; (**k**) GT100.

As depicted in Table 7, the specimens with different GT contents had different degrees of spalling (the volume change before and after failure of specimens is defined as the degree of spalling). The spalling phenomenon of GT00~GT60 was relatively light (7.9%~26.8%), concrete spalling increased in GT70 and GT80 (35.5% and 32.7%), and severe spalling was observed in GT90 and GT100 (51.1% and 54.3%). As depicted in Figure 18, most specimens had visible macroscopic cracks. Oblique shear cracks appeared in GT00, small cracks and splitting cracks were identified in GT10 and GT20, and no macroscopic cracks were found in GT30. In general, the addition of GT reduced the defects of the concrete and redistributed the force within the concrete, thus inhibiting the propagation of cracks in the concrete. However, the propagation of GT30~GT100 cracks tended to increase. GT40 had the lightest spalling, whereas two small cracks were observed in GT40. Splitting cracks were observed in GT60, and converging cracks were observed in GT80. Moreover, extensive propagation of cracks caused extensive damage to GT90 and GT100. The reason for the above result is that the GT–matrix interface, a weak part in concrete, can be separated to form interface cracks and develop into micro-cracks. Following the further action of the load, the above micro-cracks will further develop, gather, and penetrate, thus resulting in the formation of macro-cracks. Moreover, new micro-cracks appear at the tip of GT surface due to stress concentration. On one hand, this will develop into new macro-cracks; on the other hand, the original macro-crack will propagate forward, thus confirming the regularity of the compression results.

#### 3.3.2. Mesoscopic Appearance Analysis

Between the microscopic size of the nanometer level and the macroscopic size of the centimeter level, there are the microscopic sizes of the micrometer and the millimeter levels. Concrete has been considered a three-phase material consisting of aggregate at the mesoscopic level, cement stone, and the interfacial transition zone between them. Moreover, the mechanical properties of concrete can be recognized as a meso-homogeneous damage body. Concrete with different amounts of GT have different bleeding conditions, cement stone hardness, and interfacial bond strength, thus affecting the macroscopic mechanical properties of concrete [55].

**Figure 19:**
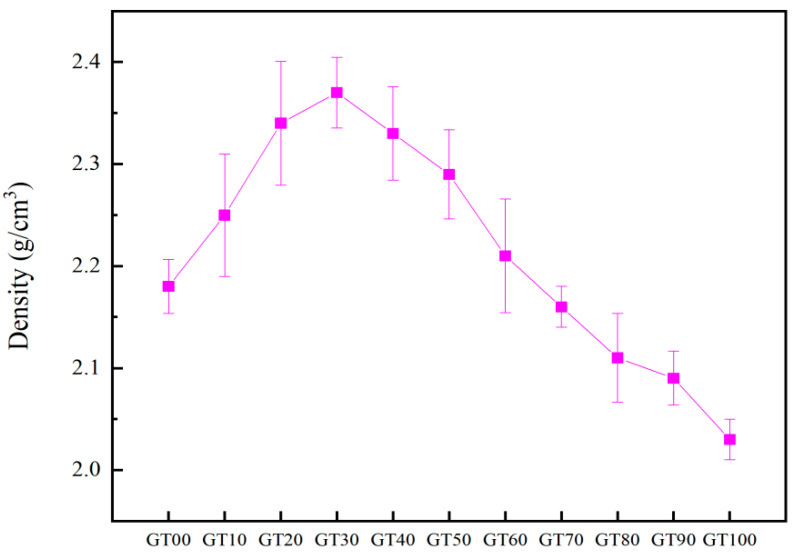
Density of specimens with different GT contents.

The specimens were dried before the flexural test. The mass and volume of the specimens were obtained by weighing and drainage methods, and the density of specimens was finally obtained (Figure 19). The dosage of 0~30% GT can increase the density of concrete. The density begins to decrease when the dosage exceeds 40%, and the dosage of 70% GT shows lower density than that of ordinary concrete, which provided the basis for the compactness of concrete with different GT contents.

**Figure 20:**
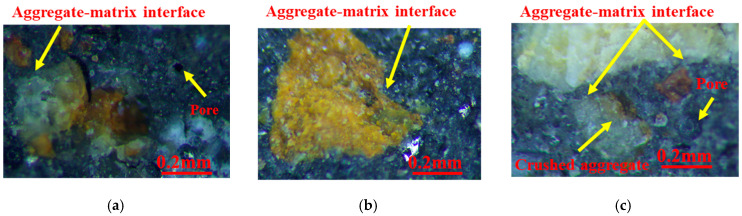

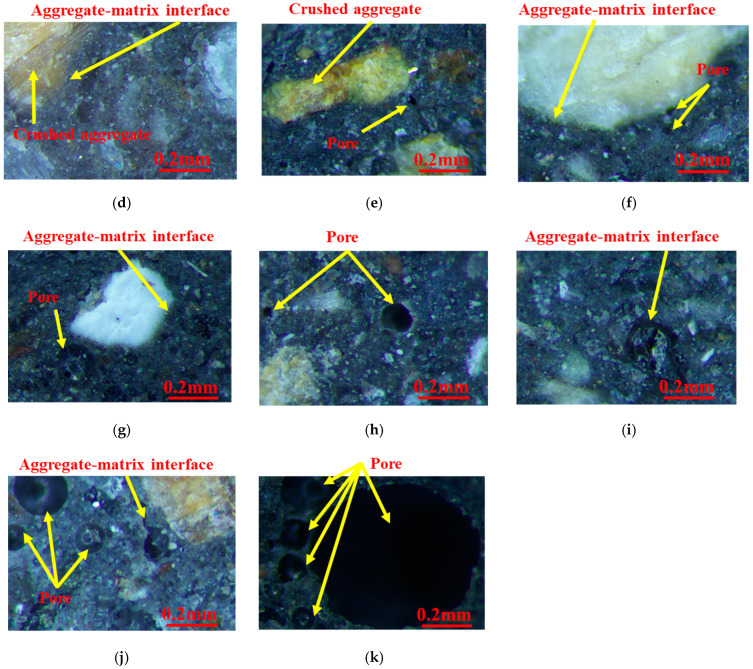
Mesoscopic appearance of graphite tailings concrete. (**a**) GT00; (**b**) GT10; (**c**) GT20; (**d**) GT30; (**e**) GT40; (**f**) GT50; (**g**) GT60; (**h**) GT70; (**i**) GT80; (**j**) GT90; (**k**) GT100.

Figure 20 presents the mesoscopic appearance of concrete after fracture. The aggregates in GT00 were well bonded to the matrix. Crushed aggregates were observed in GT20~GT40, thus suggesting that the failure of concrete was not only interface failure, but also aggregate failure, which confirmed the increase of the elastic modulus in GT20~GT40. The propagation of cracks in concrete should overcome the resistance of the aggregate and interface, which has also been confirmed by the previous flexural energy relation. The damage of GT50~GT100 basically arises from pores and interface defects. Pores appeared at the interface between the aggregate and cement stone in GT50. The interface bonding was poor in GT80 and GT90, the slurry did not fully surround the aggregate in GT80, and cracks were identified at the interface between the aggregate and the matrix in GT90. Cracks can propagate along the aggregate–matrix interface with GT content up to 90%. The poor interface bonding was attributed to the effect of GT on the effective water–binder ratio, and the coordination between GT and matrix cannot be fully exerted under stress. As GT replacement rate reached the maximum value of 100%, pore aggregation was observed on the surface of concrete, pores were attached together, and its size tended to increase. The above results showed that the interior of the specimens had a loose and porous structure, and cracks pervaded the hardened cement paste (cracks at the bond between GT and cement stone, around GT), cement stone cracks (cracks in cement paste, between GT and GT), and pores. This verifies the results shown in Figure 19.

#### 3.3.3. Environment-Friendly Evaluation of GT Concrete from the Destructive Form

Aggregate crushing was identified in GT20~GT40. 120MPa compressive strength is the minimum standard for ultra-high-performance concrete according to the Chinese standard (T/CBMF37-2018) [56], whereas all groups of concrete in this test did not have a compressive strength of 120 MPa. When GT is used to prepare ultra-high-performance ecological concrete, the crushing rate of GT might be the most significant limiting condition for the ecological properties of GT concrete. Furthermore, reducing the amount of cement can effectively reduce carbon emissions [57,58], thus if GT after special treatment can serve as cementitious material and aggregate simultaneously, the ecological properties of concrete can be significantly enhanced.

### 3.4. Mechanism Analysis of GT Replacing Sand

The production process of concrete appears to be simple, whereas it exhibits a relatively complex internal structure. Concrete contains numerous non-uniform solid phase components, as well as some closed, semi-closed, or connected pores. Concrete in this study was composed of tricalcium silicate (C_3_S), dicalcium silicate (C_2_S), tricalcium aluminate (C_3_A), and tetracalcium ferric aluminate (C_4_AF), and the internal structure of concrete changed with the GT content. The mechanism of GT replacing sand was analyzed based on the above results from the four aspects, including the wettability of aggregates, the behavior of aggregates and bubbles during stirring and vibration, the reaction mechanism of cementitious materials, as well as the interface between aggregates and the cementitious matrix.

#### 3.4.1. Analysis of Aggregate Wetting Mechanism

**Figure 21:**
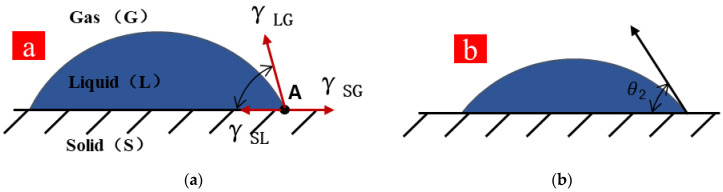
Wetting angle (**a**) for sand and (**b**) for GT.

The aggregate provides the attachment point for the reaction of the cementitious material, and cementitious material binds aggregate to form a skeleton structure, both of which promote each other. The cohesive force of cementitious paste is correlated with wetting angle, and GT are more wettable because of their rougher surface and greater water absorption. As depicted in Figure 21a, point A is the junction of fine aggregate (solid), cementitious slurry (liquid), and bubbles (gas), and point A is in a state of force balance. The force balance analysis of point A is expressed by Equation (6).

(6) $$\gamma_{SG}=\gamma_{SL}+\gamma_{LG}\cos\theta_{1}$$

where *γ*_SG_ denotes the force between the solid phase and the gas phase; *γ*_LG_ represents the force between the gas phase and the liquid phase; *γ*_SL_ is the force between the solid and liquid phases; *θ* is the wetting angle; *θ*_1_ represents the wetting angle of sand; *θ*_2_ expresses the wetting angle of GT.

Equation (7) expresses the correlation between bonding energy and surface energy.

(7) $$W_{A}=\gamma_{SG}+\gamma_{LG}-\gamma_{SL}$$

where *W_A_* is bonding work; *γ*_SG_ is the surface tension of the liquid.

(8) $$W_{A}=\gamma_{LG}(1+\cos\theta_{1})$$

Equation (8) can be obtained by Equations (6) and (7). *γ_LG_* is basically the same in concrete with different GT contents, and Equation (8) suggests that the smaller the wetting angle *θ*, the stronger the adhesion between aggregate and slurry will be (Figure 21b). When a small number of GT are added, the bonding force between aggregates will be stronger because of the smaller wetting angle of GT, the skeleton role of GT as an aggregate will be more significant. Concrete strength is affected by the internal force distribution [59]. In other words, wettability is a necessary but not sufficient condition for concrete to exhibit good adhesion, and the formation of bonding consists of the stages of rheology, diffusion, and penetration. The GT dosage was relatively suitable in this study, which was 20%~40%. GT are capable of generating more bonding force with the slurry, and a further increase of GT dosage will lead to the rheological and diffusion properties of the gel matrix to decrease. Furthermore, GT cannot be evenly distributed in concrete, and the GT skeleton cannot be fully formed, thus resulting in a decrease in the strength of the specimen.

#### 3.4.2. Mechanism Analysis of Concrete Mixing and Vibration

**Figure 22:**
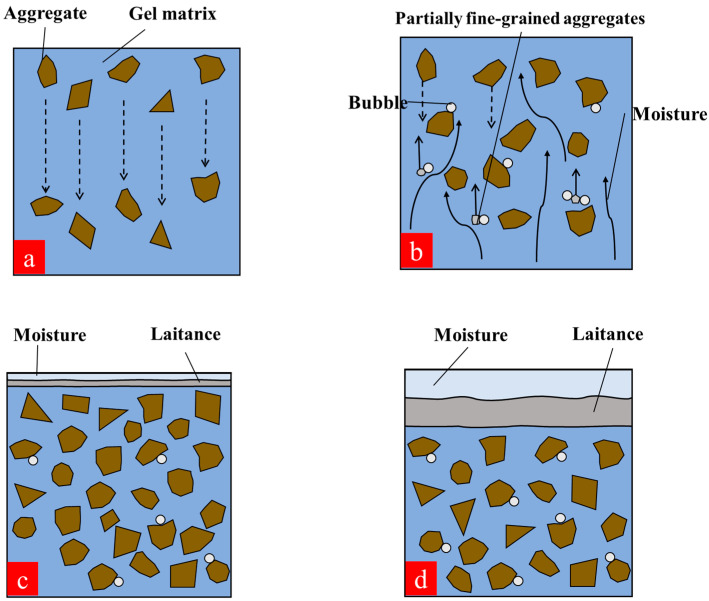
Behavior of concrete internal elements during stirring and vibration. (**a**) Aggregates sink; (**b**) fine particles and water float to the surface; (**c**) a small amount of laitance is produced; (**d**) a large amount of laitance is produced.

Vibration is considered a necessary means and step for compacting concrete. The internal friction between aggregates temporarily disappeared during the vibration process, and the concrete mixture became fluid and tended to compact under the action of gravity (Figure 22a). Moreover, the moisture rose together with some micron-sized fine aggregates (Figure 22b). The air bubbles attached to the sand surface were unstable and easily escaped during the vibration process due to the smooth surface of the sand. Different dosages of GT make the concrete skeleton systems different, and small-diameter air bubbles attached to the GT surface have difficulty escaping due to the rough surface of the GT. The GT content of nearly 30% made the GT–sand skeleton system relatively dense, and only a small amount of floating slurry was generated during the vibration process (Figure 22c). As the GT dosage was at a high value, which was 80%~100% in this study, air bubbles were easily divided into considerable small-diameter air bubbles and could all escape, and then continued to adhere to the GT surface. When the GT content was 100%, fine-grained GT were adsorbed in bubbles and rose together with bubbles, thus leading to the formation of considerable laitance (Figure 22d). In addition, coarse-grained GT will also absorb the bubbles, thus making the interface transition zone between aggregate and slurry loose and porous.

#### 3.4.3. Analysis of Reaction Mechanism of Cementitious Materials

The main component of cement is CaO, and the main chemical component of silica fume is amorphous SiO_2_. Ca(OH)_2_ after cement hydration has an orientation parallel to aggregate surfaces [60]. Numerous oriented Ca(OH)_2_ crystals exist in the aggregate–matrix interface, and the large Ca(OH)_2_ crystals parallel to aggregate surface are prone to cracking, resulting in the formation of weak regions of strength. A hydration reaction is the premise of the pozzolanic reaction, moreover, the pozzolanic reaction eliminates the negative effect of the hydration reaction and provides a guarantee for the strength of concrete [61,62,63,64]. As GT dosage arrives at different values, the adhesion of cementitious material to aggregate will be affected.

**Figure 23:**
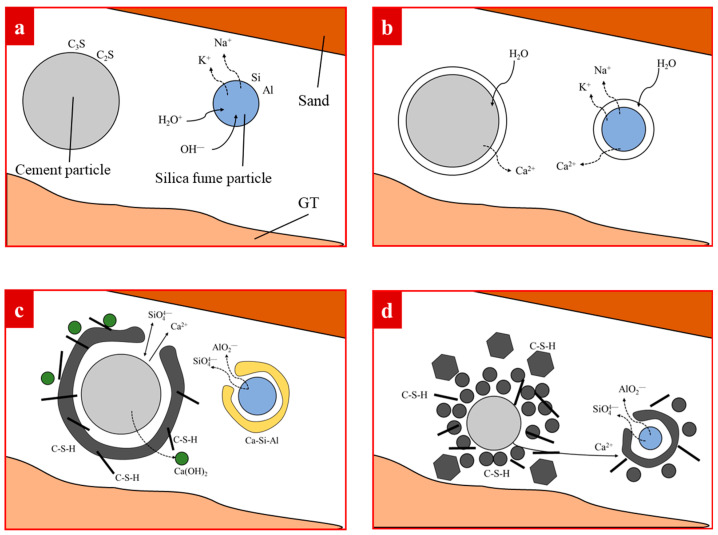

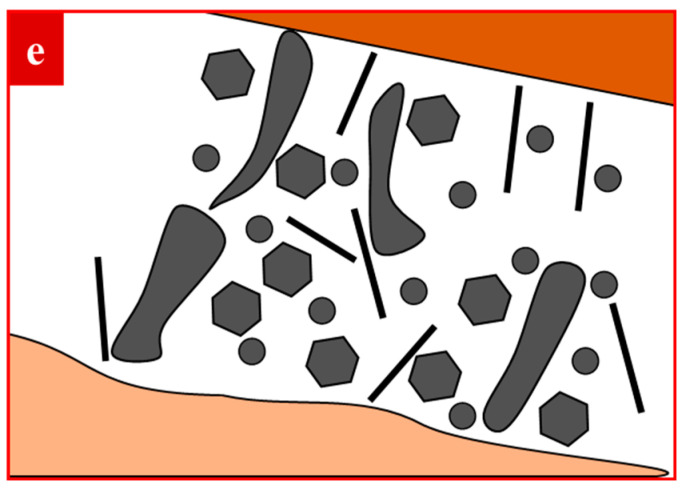
The hydration mechanism of cementitious materials (C_3_S—Tricalcium Silicate, C_2_S—Dicalcium Silicate, C–S–H—Calcium Silicate Hydrate). (**a**) Film formation; (**b**) cementitious material absorbs water in the GT–sand system; (**c**) C–S–H gel is gradually formed in the GT–sand system; (**d**) cement and silica fume particles are consumed, producing C–S–H; (**e**) a large number of C–S–H gels are formed in the GT–sand system.

Cement hydration makes GT and sand form skeleton structures, and silica fume refine the matrix–aggregate interface [65]. Hydration of cementitious materials is significantly correlated with the GT–sand system. The reaction went through five steps (Figure 23). At Stage 1, C_3_S and C_2_S formed thin films on the surface of cement particles, and Si and Al were enriched to form a thin film on the surface of silica fume (Figure 23a). At Stage 2, the electrostatic force of ions destroyed the original water structure and formed a layer of water molecules around it. Cement and silica fume particles absorbed water in the GT–sand system (Figure 23b). Cementitious particles took GT as nucleation points and adhered to the surface of GT. GT and sand were gradually connected to form a system, and the two promoted each other. The higher the content of GT, the more water loss in the slurry was, thus resulting in a decrease in the dissolution rate of ions. At Stage 3, C_3_S and C_2_S were hydrated to form C–S–H and Ca(OH)_2_. Ca–Si–Al film was formed on the surface of silica fume (Figure 23c). Ca/Si plays an important role in the hardening process of concrete and can affect the degree of polymerization of silicates [36,66]. Hydration and pozzolanic reaction rates are not fully consistent, leading to the Ca/Si change in the slurry with GT content. Excess GT may adsorb silica fume particles and reduce the generation of C–S–H due to the small amount of silica fume. At Stage 4 and Stage 5, C–S–H tended to fill the entire slurry and combined with the GT–sand system to form a concrete structure (Figure 23d,e).

#### 3.4.4. Aggregate–Cementitious Matrix Interface Mechanism Analysis

**Figure 24:**
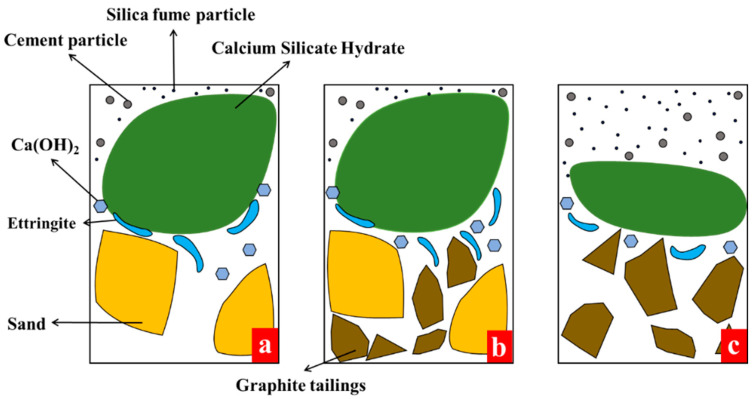
Schematic diagram of the aggregate–matrix interface. (**a**) Aggregate skeleton system without GT; (**b**) reinforcing effect of an appropriate amount of GT on the aggregate framework system; (**c**) weakening effect of high content of GT on the aggregate framework system.

As mentioned earlier, the gaps between the sand were large and could be filled with each other well in GT00 (Figure 24a). GT could fill the gaps by adding an appropriate amount of GT (Figure 24b), thus lengthening the path for the crack to propagate and making the crack require more energy to penetrate the concrete. Nevertheless, the particle size distribution of sand tended to increase first and then decrease, with good particle size distribution. GT are rough and angular, with an irregular particle size distribution. GT particles were poorly packed in GT100 (Figure 24c). On one hand, the matrix was prone to stress concentration on the GT surface when concrete was stressed. On the other hand, GT affected the hydration reaction because of its high water absorption. The hydration reaction of the gelled particles penetrated from the surface to the interior, and the unhydrated gelling materials were clumped together, reducing the effective area involved in the reaction [67]. As a result, the C–S–H decreased.

#### 3.4.5. Environment-Friendly Evaluation on the Mechanism of GT Replacing Sand

Replacing sand with tailings needs to fully consider the physical characteristics of the tailings themselves. The mechanism analysis of GT replacing sand can provide research ideas for other tailings-based environment-friendly concrete and maximize the ecology of concrete.

## 4. Conclusions

This study successfully prepared the environment-friendly concrete by using GT and sand as fine aggregates. To reveal the influence of GT on mechanical properties of concrete, a comprehensive study has been examined, and the following conclusions are drawn.

(1) In general, GT and sand have similar chemical compositions, which lays a basis for GT to replace sand. Nevertheless, the differences in water absorption, crush value, and roughness make GT and sand have different mechanisms.
(2) There is a threshold for the effect of GT content on the mechanical strength of concrete. Compared to GT00, the compressive strength and elastic modulus of GT30 increased by 12.68% and 96.42%, respectively, and the compressive strength and elastic modulus of GT100 decreased by 5.37% and 25.96%, respectively. The filling effect of GT on sand can inhibit the propagation of cracks and increase the energy required for concrete failure. A rate of 30% is recommended as the optimal GT replacement rate.
(3) There are significant differences in concrete spalling degree (GT00~GT100). GT30 and GT40 have the lowest spalling degree (11.3% and 7.9%), and GT90 and GT100 have the largest spalling degree (51.1% and 54.3%). In the mesoscopic analysis, crushed aggregates are observed in the damaged specimens of GT20, GT30, and GT40, obvious defects were identified at the aggregate–matrix interface in GT80 and GT90, and numerous pores were observed in GT100. The above results suggest that the incorporation of an appropriate amount of GT can increase the bonding ability at the interface, and the skeleton effect of GT is significantly reduced when GT achieve the maximum value of 100%.
(4) The characteristics of high water absorption, unreasonable particle size distribution, and large crushing value limit the feasibility of GT completely replacing sand. However, the wetting angle of GT is smaller, which is a good phenomenon for the bonding of GT to the matrix. There are still many places worth studying to increase the amount of GT.

The results of this study can provide a reference for the mechanical mechanism of GT to prepare environment-friendly concrete. In the future, the amount of GT can serve as the basis for the ecological evaluation of concrete. Moreover, the experimental results of mechanical properties and durability can lay a basis for the feasibility evaluation of GT to replace sand. The correlation between GT content, mechanical properties, and durability can be developed, and the optimal solution can be applied to practical engineering.

## Figures and Tables

**Table 1:** Chemical composition of cement and silica fume.

Materials	SiO_2_	CaO	Al_2_O_3_	Fe_2_O_3_	MgO	K_2_O	LoI
Cement/%	24.66	55.46	7.09	2.71	2.15	0.65	7.28
Silica fume/%	92.8	0.31	0.76	0.52	0.53	2.2	2.88

**Table 2:** Basic physical properties of sand and GT.

	Bulk Density /(kg/m^3^)	Apparent Density /(kg/m^3^)	Fineness Modulus	Crush Value/%	Porosity /%	Water Absorption Rate /%
Sand	1572	2588	2.2	15.04	39.38	0.48
GT	1454	2779	1.1	28.59	47.84	1.77

**Table 3:** Chemical composition of sand and GT.

Mineral Composition		SiO_2_	CaO	Al_2_O_3_	Fe_2_O_3_	MgO	K_2_O	LoI
Proportion/%	Sand	69.84	8.27	7.39	2.95	2.15	1.44	7.96
	GT	56.64	13.34	11.52	7.25	3.72	3.54	3.99

**Table 4:** Comparison of particle sizes of sand and GT.

Particle Size (mm)	2.50~1.25	1.25~0.63	0.63~0.315	0.315~0.150	0.150~0
Sand	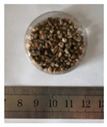	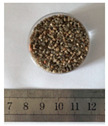	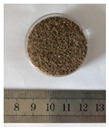	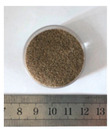	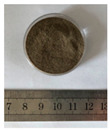
Proportion/%	13.62	24.56	39.96	16.93	4.93
GT	--	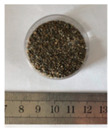	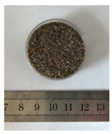	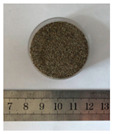	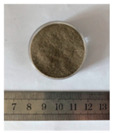
Proportion/%	--	2.19	28.23	28.27	41.32

**Table 5:** Mesosurface of GT and sand.

Particle Size (mm)	2.5~1.25	1.25~0.63	0.6~0.315	0.315~0.15	0.15~0
Sand	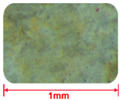	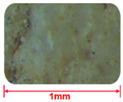	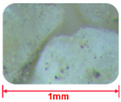	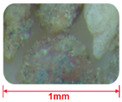	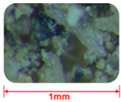
GT	——	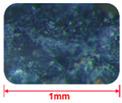	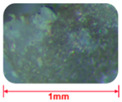	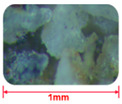	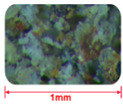

**Table 6:** Test mix ratio (kg/m^3^).

Group	Water	Cement	Silica Fume	Sand	GT	Admixture
GT00	201.03	804.12	201.03	1206.18	0	17.09
GT10				1085.56	120.44	17.09
GT20				964.94	241.24	17.09
GT30				844.33	361.85	17.09
GT40				723.71	482.47	17.09
GT50				603.9	603.9	17.09
GT60				482.47	723.71	17.09
GT70				361.85	844.33	17.09
GT80				241.24	964.94	17.09
GT90				120.44	1085.56	17.09
GT100				0	1206.18	17.09

**Table 7:** Concrete spalling degree.

	GT00	GT10	GT20	GT30	GT40	GT50	GT60	GT70	GT80	GT90	GT100
Spalling Degree (%)	26.8	19.6	16.8	11.3	7.9	15.4	17.2	35.5	32.7	51.1	54.3

## Data Availability

Data derived from the current study can be provided to readers upon request.
